# Improvement in the Assessment of Response to Preoperative Chemoradiotherapy for Rectal Cancer Using Magnetic Resonance Imaging and a Multigene Biomarker

**DOI:** 10.3390/cancers13143480

**Published:** 2021-07-12

**Authors:** Eunhae Cho, Sung Woo Jung, In Ja Park, Jong Keon Jang, Seong Ho Park, Seung-Mo Hong, Jong Lyul Lee, Chan Wook Kim, Yong Sik Yoon, Seok-Byung Lim, Chang Sik Yu, Jin Cheon Kim

**Affiliations:** 1 Asan Medical Center, Department of Colon and Rectal Surgery, University of Ulsan College of Medicine, Seoul 05505, Korea; cec1103@amc.seoul.kr (E.C.); iamleejong@amc.seoul.kr (J.L.L.); crscwkim@amc.seoul.kr (C.W.K.); yoonys@amc.seoul.kr (Y.S.Y.); sblim@amc.seoul.kr (S.-B.L.); csyu@amc.seoul.kr (C.S.Y.); jckim@amc.seoul.kr (J.C.K.); 2 Department of General Surgery, Good Morning Hospital, Pyeongtaek-si 17874, Korea; deswoo16@gmail.com; 3 Asan Medical Center, Department of Radiology, University of Ulsan College of Medicine, Seoul 05505, Korea; jongkeon.jang@amc.seoul.kr (J.K.J.); seongho@amc.seoul.kr (S.H.P.); 4 Asan Medical Center, Department of Pathology, University of Ulsan College of Medicine, Seoul 05505, Korea; shong28@amc.seoul.kr

**Keywords:** locally advanced rectal cancer, preoperative chemoradiotherapy, radiation prediction index, magnetic resonance imaging tumor regression grade, pathologic tumor regression grade, combined response prediction value

## Abstract

**Simple Summary:**

Preoperative chemoradiotherapy is now the gold standard for treating locally advanced rectal cancer and has been demonstrated to decrease local recurrence and promote sphincter preservation. Therefore, developing methods to accurately predict patients’ response to chemoradiation is imperative for choosing the best surgical option after chemoradiation and predicting the oncologic outcomes of patients. Radiological tools and endoscopy are the most commonly used tools for post-treatment response assessment. In addition, examining the expression levels of genes correlated with treatment response could provide clinicians with more power to gauge each patient’s potential to respond. In this study, we explored how biological and radiological tools can be used together to provide a more tailored and multidimensional representation of patients’ response status.

**Abstract:**

The response to preoperative chemoradiotherapy (PCRT) is correlated with oncologic outcomes in patients with locally advanced rectal cancer. Accurate prediction of PCRT response before surgery can provide crucial information to aid clinicians in further treatment planning. This study aimed to develop an evaluation tool incorporating a genetic biomarker and magnetic resonance imaging (MRI) to improve the assessment of response in post-CRT patients with locally advanced rectal cancer. A total of 198 patients who underwent PCRT followed by surgical resection for locally advanced rectal cancer between 2010 and 2016 were included in this study. Each patient’s response prediction index (RPI) score, a multigene biomarker developed in our previous study, and magnetic resonance tumor regression grade (mrTRG) score were added to create a new predictive value for pathologic response after PCRT, called the combined radiation prediction value (cRPV). Based on the new value, 121 and 77 patients were predicted to be good and poor responders, respectively, showing significantly different cRPV values (*p* = 0.001). With an overall predictive accuracy of 84.8%, cRPV was superior to mrTRG and RPI for the prediction of pathologic chemoradiotherapy response (mrTRG, 69.2%; RPI, 77.3%). In multivariate analysis, cRPV was found to be the sole predictor of tumor response (odds ratio, 32.211; 95% confidence interval, 14.408–72.011; *p* = 0.001). With its good predictive value for final pathologic regression, cRPV may be a valuable tool for assessing the response to PCRT before surgery.

## 1. Introduction

The effectiveness of preoperative chemoradiotherapy (PCRT) in treating locally advanced rectal cancer (LARC) has opened a dialog regarding whether radical resection, long believed to be the gold standard surgical strategy, is truly required for all patients with comparable oncologic outcomes [1]. Recently, in addition to the non-inferior results in patients with stage I rectal cancer who have undergone neoadjuvant chemoradiation followed by local excision [2], a prospective study found that patients with LARC also showed results comparable to those who have undergone radical resection after active surveillance and local excision, given that the patients showed an endoluminal response to PCRT [3]. A recently concluded five-year randomized trial found no difference in oncological outcomes between local excision and total mesorectal excision in patients with T2–3 LARC who showed good clinical response [4], further corroborating this point. Deferral of surgery for patients whose clinical evaluations indicate complete remission is also being considered [5,6,7,8]. In light of this trend for avoiding radical resection and its possible morbidities, more attention needs to be paid as to whether our clinical assessment of treatment response is truly reliable.

While magnetic resonance imaging (MRI) is the most widely used tool for the evaluation of response after PCRT, its pT and pN stage predictability is limited. A Dutch study [9] retrospectively reviewed patients who underwent R0 resection after PCRT and found that three independent MRI readers correctly predicted the ypT stage in 47–68% of patients and ypN stage in 68–70% of patients. However, they correctly predicted ypT and N stages combined in only 28–47% of patients. The more recently proposed magnetic resonance tumor regression grade (mrTRG) is a valuable tool for assessing PCRT response; it uses tumor characteristics and degree of fibrosis, similar to the pathologic tumor regression grade (pTRG) system, to clinically evaluate the extent of regression [10,11,12]. However, mrTRG has limitations. A British group comparing mrTRG and pTRG of patients with rectal cancer from two independent phase II trials (EXPERT and EXPERT-C) found positive and negative predictive values of mrTRG for the prediction of pTRG-based complete regression of 36.8% and 89.4%, respectively, despite a high inter-observer concordance rate of 92.0% [13].

Using molecular biomarkers could provide valuable information on how genetically inclined each patient is to respond to PCRT [14]. Some scholars, including a Korean group [15], have found a correlation between PCRT response and expression levels of certain cancer-related mRNAs, while other scholars have found the same with certain miRNAs [16,17]. The radio-response prediction index (RPI) is a part of such efforts. The multigene biomarker model is a system that calculates a genetic score for each patient, which can then be used to assess the likelihood of a patient responding to PCRT. It was developed by extracting mRNA from PCRT formalin-fixed paraffin-embedded tumor tissues and sorting for cancer regulation genes that showed differential expression levels according to the degree of response [18].

In this study, we combined RPI, a genetic biomarker, with mrTRG, a clinical evaluation value of regression, to improve the efficacy of assessing tumor response to PCRT.

## 2. Materials and Methods

### 2.1. Study Population

Patients with mid- and low-rectal cancer (lesions located within 10 cm of the anal verge) who underwent PCRT followed by surgical resection between January 2010 and December 2016 were enrolled. This retrospective study protocol was approved by the institutional review board of the Asan Medical Center. Patients who did not receive surgical treatment, those who did not have pretreatment biopsy specimens, those who did not undergo post-treatment MRI, and those who could not be assessed for post-treatment pathological response were excluded from the study. Ultimately, 198 patients were enrolled in the study.

### 2.2. PCRT and Surgery

The PCRT doses were 45–50.4 Gy in 25 or 28 fractions. The PCRT regimen comprised two cycles of intravenous 5-fluorouracil (5-FU; 375 mg/m^2^/day) plus leucovorin (LV; 20 mg/m^2^/day) or oral capecitabine (1650 mg/m^2^/day). 5-FU and leucovorin were delivered in a bolus format over three days at the first and fifth weeks of radiation therapy (RT), and oral capecitabine (1650 mg/m^2^/day) was administered twice daily during RT. Almost all patients underwent radical resection (including total mesorectal excision) 5–12 weeks after completing PCRT. Selected patients who were assessed to have achieved total or near-total regression underwent full-thickness local excision.

### 2.3. Clinical Assessment of Tumor Response after PCRT

Each patient underwent MRI 4–6 weeks after the completion of PCRT; 1.5-T or 3.0-T scanners (modes: MAGNETOM Avanto and Skyra from Siemens Medical Solutions) were used to create high-resolution images of 0.5–0.7-mm pixel size and 3-mm thickness with no interslice gap, resulting in T-2 weighted, fast spin-echo images in axial, coronal, sagittal, and oblique planes as well as axial diffusion-weighted images (ß factors: 0–1000 s/mm^2^). Two experienced radiologists, each with at least five years of training in the field of abdominal radiology, separately evaluated each patient’s images to apply a five-tier mrTRG as follows: mrTRG 1, complete regression (absence of tumor signal and barely visible treatment-related scar); mrTRG 2, good regression (predominantly low signal intensity fibrosis with no obvious residual tumor signal); mrTRG 3, moderate regression (predominantly low signal intensity fibrosis with obvious areas of intermediate signal intensity); mrTRG 4, slight regression (limited areas of low signal intensity fibrosis or mucin but mostly tumor); and mrTRG 5, no regression (intermediate signal intensity or the same appearance as the original tumor). When the mrTRG score was incongruent between the two evaluators, a third radiologist was invited to review the images, and the score chosen by the majority was selected as the final score for the patient.

### 2.4. Pathologic Assessment of Tumor Response after Surgery

Hematoxylin and eosin-stained surgical specimens were evaluated by pathologists who were highly experienced in gastrointestinal pathology according to the pTRG guidelines published by the Gastrointestinal Pathology Study Group of the Korean Society of Pathologists [19]. The specimens were classified as follows: “complete” (no residual tumor cells), “near-complete” (abundant fibrosis with only a few or scattered tumor cells), “partial” (easily identifiable residual tumor gland in tumor bed), or “poor or none” (tumor cells did not demonstrate any response to chemoradiotherapy because of the presence of abundant residual adenocarcinoma). Patients were finally classified as “good responders” if their specimens showed complete or near-complete regression or as “poor responders” if their specimens showed less regression.

### 2.5. Development and Evaluation of the Combined Response Prediction Value

Each patient’s mrTRG score in reverse order was added to their RPI value to create a new value called the combined response prediction value (cRPV). For example, if a patient showed “good regression” on MRI after PCRT (mrTRG score = 2), 3 would be added to the patient’s RPI value to obtain the cRPV value. The cRPV value was then evaluated in comparison with the mrTRG value for the prediction of a patient’s pathologic response to PCRT, as represented by the pTRG value.

### 2.6. Statistical Analysis

The clinicopathologic characteristics were analyzed using Chi-square and independent-sample *t*-tests, as well as logistic regression. Categorical variables were compared using the Chi-squared test, and Student’s *t*-tests were used to analyze normally distributed continuous data. All continuous variables are presented as a median (interquartile range).

Receiver operating characteristic (ROC) curves were used to evaluate the ability of cRPV to predict good responders to PCRT. The value of cRPV that yielded the highest Youden’s J index (1.917) was used as the cutoff value for cRPV. A value below this cutoff was considered a poor response, whereas a value above this cutoff was considered a good response. The same was followed for RPI (cutoff value, −0.005). For mrTRG, patients who received mrTRG 1 or 2 were considered to be good responders, while those who received mrTRG 3–5 were considered poor responders. Multivariate logistic regression analysis was used to measure the association between clinicopathological features and pathologically good response. Moderated regression analysis was used to detect any impact the interval between PCRT and restaging MRI may have on the prediction of final pathology by cRPV using various intervals patients had as the mediating variable. 

Differences were considered statistically significant at *p*-values < 0.05. Statistical analyses were performed using IBM SPSS software (version 23.0; IBM Corp., Armonk, NY, USA) and STATA version 16 (STATA Corp., College Station, TX, USA).

## 3. Results

### 3.1. Patient Characteristics

A total of 198 patients were enrolled in the study (126 men, 63.6%). The median age was 61 years (range, 32–89 years). On pre-PCRT clinical staging, 176 patients (88.9%) were suspected to have metastatic lymph nodes. A total of 118 (59.6%) patients received capecitabine for chemotherapy. Most patients underwent radical resection (186, 93.9%), and 12 (6.1%) patients underwent local excision. The mean interval from completion of PCRT to surgical resection was 52 days (range, 35–82 days). Pathologic examination revealed that 83 (41.92%) patients had ypT3+ disease and 46 (23.2%) had metastatic lymph nodes. According to the pTRG criteria, 113 (57.1%) patients were classified as having complete or near-complete pathologic regression (Table 1).

### 3.2. Post-PCRT Response Assessment by cRPV

**Figure 1:**
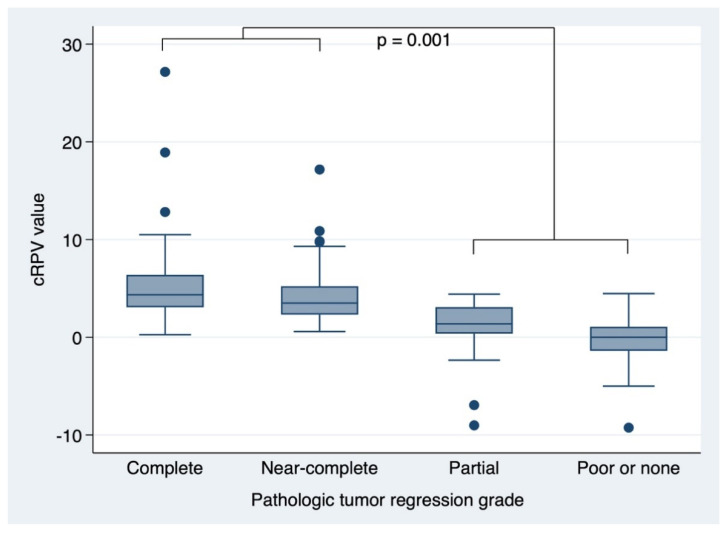
cRPV distribution boxplots according to the pathologic response to preoperative chemoradiotherapy. cRPV, combined response prediction value.

The cRPV score was significantly higher in patients with complete or near-complete regression than in those with partial or poor/no regression (median value: 4.9448 vs. 0.5229, *p* = 0.001; Figure 1).

**Figure 2:**
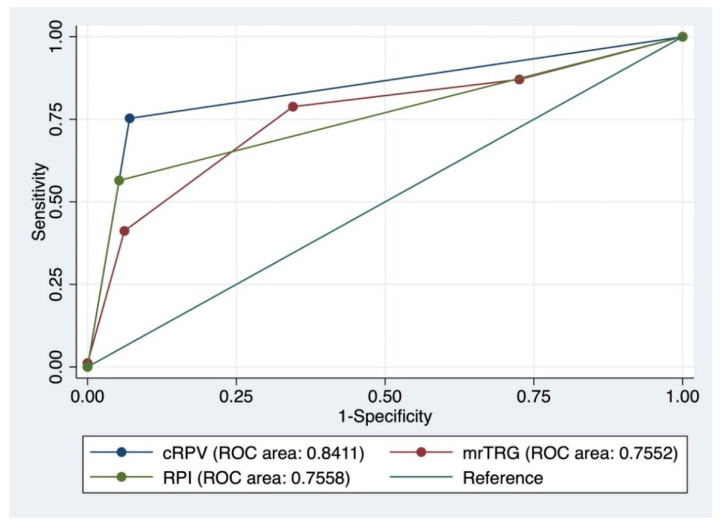
Receiver operating curve of mrTRG and cRPV in comparison with RPI in predicting pathologic tumor regression grade. mrTRG, magnetic resonance tumor regression grade; cRPV, combined response prediction value; RPI, radiation prediction index.

According to the cRPV, 121 (61.1%) and 77 (38.9%) patients were good and poor responders, respectively. An ROC curve drawn for the cRPV for the prediction of pathologic good and poor responders yielded an area under the curve (AUC) of 0.8411 (95% confidence interval [CI], 0.787–0.892; Figure 2).

The cRPV showed a sensitivity, specificity, positive predictive value, and negative predictive value of 90.3%, 77.6%, 84.3%, and 85.7%, respectively, yielding a higher predictive accuracy of 84.8% compared to that of mrTRG or RPI alone (69.2% and 78.3%, respectively) (Table 2).

An ROC curve drawn for comparison showed a larger AUC for cRPV than for mrTRG or RPI alone (AUC: 0.7552 [95% CI, 0.686–0.824] and 0.7558 [95% CI, 0.699–0.813], respectively). To evaluate the predictive accuracy of cRPV, other factors that may influence the response to radiotherapy were analyzed. In multivariate analysis, cRPV was the only significant predictor of a good response to PCRT (odds ratio [OR] 32.211, 95% CI 14.408–72.011, *p* = 0.001; Table 3).

In the moderated regression analysis, the coefficient of determination (R2) of the cRPV was determined to be 0.502, and the interaction variable (cRPV × interval between completion of PCRT and follow-up MRI in days) was found to have no significant impact on the pathologic regression (*p* = 0.241) (Table S2). No specific number of days spent until restaging MRI after the end of PCRT proved the best fit for cRPV prediction of pathologic outcome.

**Figure 3:**
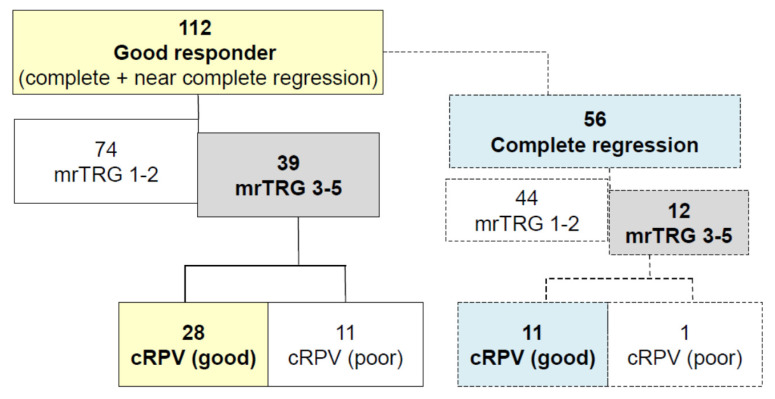
Prediction of good responders with cRPV. cPRV can detect 71.8% of patients who were assessed as clinically poor responders but eventually were considered good responders. mrTRG, magnetic resonance tumor regression grade; cRPV, combined response prediction value.

Among the pathologic good responders (*n* = 112), 39 (34.8%) were wrongly assessed as clinical poor responders with MRI (mrTRG3–4). Among these 39 patients, cRPV detected 28 (28/39, 71.8%) pathologic good responders. MRI incorrectly assessed 12 patients with pathologic complete regression as clinical mrTRG3-. Among 12 incorrectly assessed patients, 11 patients (11/12, 91.7%) could be corrected for response assessment with cRPV (Figure 3). Patients with mrTRG1–2 showed same results with cRPV in patients with pathologic good responders. Pathologically poor responders were wrongly assessed as clinical good responder as mrTRG1–2 in 18 (20.9%) patients. The cRPV would detect 5 (27.8%) real poor responder among these 18 patients. 

With regard to the ability of cRPV to predict the pathologic response to PCRT, there was no difference between patients for whom the prediction was correct (“Concordant,” *n* = 168) and those for whom the prediction was incorrect (“Discordant,” *n* = 30) in any clinical factor except for pre-PCRT CEA levels (median value: 2.1 vs. 2.15 ng/mL (*p* = 0.038), pTRG (*p* = 0.001) and ypT stage (*p* = 0.0433). The discordant group had more poor responders (63.3%) than did the concordant group (39.3%), but this difference was not significant (Table 4).

Both mrTRG and cRPV incorrectly predicted the pathologic results in 24 patients. These patients showed a tendency toward a low rate of total regression (4.2%) and a larger proportion of high ypT stages (20%, 33%, and 45% for ypT0–1, ypT2, and ypT3, respectively; Table S1).

## 4. Discussion

According to our data, cRPV which combines MRI results with genetic biomarker showed improved predictive values (both positive and negative) compared to mrTRG alone. Even when adjusted for other factors that may be related to radio-responsiveness using multivariate analysis, cRPV showed a high level of predictability.

Our study is unique as this study combine a genetic biomarker with clinical imaging to assess treatment response in LARC. In practice, we are hopeful that cRPV will provide physicians with additional information in determining the optimal surgical strategy for post-PCRT patients, especially by allowing them to discern patients who are expected to respond poorly to treatment and not to recommend organ-preserving strategies to them.

Despite many studies favoring the ability of MRI to depict pre- and post-PCRT rectal cancer status [20,21], depending on MRI for choosing surgical strategies in post-PCRT patients raises concerns. The mrTRG has recently been widely used to evaluate clinical tumor response to chemoradiotherapy in patients with LARC treated with PCRT. However, the agreement between mrTRG and pathologic regression is not satisfactory enough [9,13]. Smith et al. [22] reported a substantially poorer five-year survival for clinical good responders following a wait-and-watch approach, who later underwent salvage surgery if recurrence was noted, than for clinically good responders who received radical resection after PCRT regardless of whether they had a pathologic complete response. This study highlighted a category of patients for whom imaging assessment failed to represent disease status and provided insufficient operative strategies, thus resulting in a shorter lifespan and mentioned importance of clinical response evaluation

Although studies have combined endoscopic findings and physical examination with mrTRG to improve the accuracy of response assessment after PCRT, inter-observer variation is a limitation of these evaluation method [23,24]. The cRPV could help in selecting non-operative surgical strategies after PCRT in patients with indeterminate post-PCRT MRI findings. For example, we would try deferral of surgery for patients with cRPV, indicating good responders with confusing results of mrTRG between good and poor response. Therefore, cRPV may help determine post-PCRT treatment. In the present study, we identified 91.7% of patients with pathologic total regression who were wrongly assessed as poor responders using MRI. Therefore, genetic biomarkers can be used as complementary imaging biomarkers for response assessment. For poor responders, however, cRPV can provide additional optimal information in 27.8% of patients who were assessed as good responders with MRI. Therefore, cPRV would be more beneficial in assessing patients who are supposed to have a good response and can be useful in detecting patients who may receive an organ-preserving approach. Clinical applications of cRPV need to be evaluated prospectively.

The timing of surgery for post-PCRT patients has been debated. Some studies have favored early surgery (approximately 6 weeks after the end of chemoradiation), while other studies have favored delayed surgery (>12 weeks after the end of chemoradiation) to maximize the chance of complete regression [25,26,27,28,29]. Ideally, restaging MRI would be the most informative if performed at a time when PCRT has had a maximal effect on the tumor regression. In a recent study, Seo et al. [30] observed a sequentially increasing tumoricidal effect of chemoradiation starting at 4 weeks, continuing up to 22 weeks after treatment. They proposed delaying MRI until surgery was imminent. In the present study, interval between restaging MRI after completing PCRT was not different between those whose pathologic response were correctly or incorrectly predicted by cRPV (Table 4, 41 vs. 43 days; *p* = 0.489). However, differences in the interval to MRI evaluation between the two groups were not long enough to make a conclusion. Moderated regression analysis was performed to find interval that best results in cRPV’s prediction of pathologic response failed. Within the examined range of interval between completion of PCRT and post-PCRT MRI assessment in this study (median 41–43 days), cPRV is useful in detecting good responders. However, we need to confirm the utility of cPRV according to the timing of MRI evaluation for a longer time in patients with non-operative management strategies.

Furthermore, the ypT stage tended to be higher for patients for whom the prediction using cRPV was incorrect; however, the initial or post-PCRT clinical stage did not differ between patients for whom the prediction using cRPV was incorrect vs. correct. A similar pattern was observed for patients for whom the prediction using cRPV and mrTRG was incorrect, leading to the speculation that mrTRG, rather than RPI, may be more responsible for this error. Our study design accounted for interobserver variability by inviting a third experienced radiologist. One possible explanation for the discrepancy in pathologic and imaging assessment is the chance of microscopic viable residual cancer in fibrotic tissues that appear with decreased signal intensity on T2-weighted images, which is approximately 50% [31]. 

The present study has several limitations. Several patients in the study population received PCRT at other hospitals close to their residences. While we ascertained that these treatments adhered to the chemoradiation protocol above, the brevity of outside medical records limits current researchers in knowing of any variation in detailed radiation techniques or fields, if any. Second, RPI was developed using the PanCancer Pathway kit, which may preclude as-yet-undiscovered biomarkers that are significantly related to chemoradiation response. Despite these limitations, our study is the first to combine rather than compare the genetic and clinical aspects of response to PCRT, with easily reproducible methods of sample collection, analysis, and interpretation. 

## 5. Conclusions

We developed cRPV as a guidance tool that combines the genetic profile of each patient’s tumor with its likelihood of responding to PCRT, with the tumor regression grade indicated by mrTRG. Owing to its good predictive value for pathologic tumor regression, cRPV can be used to develop more effective treatment plans for patients with LARC.

## Figures and Tables

**Table 1:** Clinicopathologic characteristics of patients (*n* = 198).

Characteristics	No (%) or Median [Range]
Age, years	61 (32–89)
Sex	
Female	72 (36.4)
Male	126 (63.6)
Tumor location from AV, cm	4.0 (0.3–10)
Pre-PCRT CEA, ng/mL	2.2 (0.47–39)
Pre-PCRT cT stage	
cT3	176 (88.9)
cT4	22 (11.1)
Chemotherapeutic regimen with radiotherapy	
Capecitabine	118 (59.6)
5-fluorouracil/leucovorin	80 (40.4)
Radiation dose, Gy	50.4 (43.2–50.4)
Post-PCRT CEA, ng/mL	2.6 (0.47–39)
Interval between completion of PCRT and surgical resection, days	53 (35–82)
Operative method	
Local excision	12 (6.1)
Radical resection	186 (93.9)
Pathologic Tumor regression grade of primary tumor	
Complete	56 (28.3)
Near-complete	57 (28.8)
Partial	50 (25.3)
Poor or none	35 (17.7)
Post-operation T stage (pathologic)	
ypT0	55 (27.8)
ypT1–2	60 (30.3)
ypT3+	83 (41.9)
Post-operation N stage (pathologic)	
ypNx	12 (6.1)
ypN (−)	140 (70.7)
ypN (+)	46 (23.2)

AV = anal verge; PCRT = preoperative chemoradiotherapy; CEA = carcinoembryonic antigen.

**Table 2:** Comparison of mrTRG, RPI, and cRPV in predicting pathologic good response (complete or near-complete regression).

Predictability	cRPV (%)	mrTRG (%)	RPI (%)
Sensitivity	90.3	65.5	94.7
Specificity	77.6	78.8	56.5
Positive predictive value	84.3	80.4	74.3
Negative predictive value	85.7	63.2	88.9
Predictive accuracy	84.8	69.2	78.3

mrTRG, magnetic resonance imaging tumor regression grade; RPI, radiation prediction index; CRPV, combined response prediction value.

**Table 3:** Factors associated with good responders (multivariate analysis).

Variable	Odds Ratio	95% Confidence Interval	*p*-Value
Sex			0.615
Female	1		
Male	1.242	0.533–2.894	
Clinical T stage			0.357
cT3	1		
cT4	1.848	0.500–6.827	
Clinical N stage			0.967
cN(−)	1		
cN(+)	0.974	0.273–3.466	
Interval between completion of PCRT and surgical resection (weeks)			0.297
≤8	1		
>8	0.614	0.246–1.534	
Pre-PCRT CEA (ng/mL)			0.13
≤6	1		
>6	2.208	0.791–6.163	
cRPV			0.001
Predicted poor responder (<1.917)	1		
Predicted good responder (≥1.917)	32.311	14.408–72.011	

**Table 4:** Clinicopathologic characteristics of patients for whom the prediction was incorrect according to cRPV.

Variables	No (%) or Median [Range]		*p*-Value
	Concordant (*n* = 168)	Discordant (*n* = 30)	
Age (years)	61 (36–89)	63.5 (32–86)	0.299
Sex			0.708
Female	62 (36.9)	10 (33.3)	
Male	106 (63.1)	20 (66.7)	
Tumor location from AV, cm	4.0 (0.3–10)	5.0 (1–10)	0.832
Pre-PCRT CEA, ng/mL	2.1 (0.3–115)	2.15 (0.39–11.3)	0.038
Pre-PCRT clinical stage			
Clinical T stage			0.4
cT3	148 (84.1)	28 (93.3)	
cT4	20 (11.9)	2 (6.7)	
Clinical N stage			0.674
cN(−)	18 (10.7)	4 (13.3)	
cN(+)	150 (89.3)	26 (86.7)	
Radiation dose, Gy	50.4 (43.2–50.4)	50.4 (45–50.4)	0.734
Post-PCRT CEA, ng/mL	1.6 (0.47–39)	1.5 (0.85–4)	0.512
Interval between completion of PCRT and follow-up MRI, days	41 (17–75)	43 (30–55)	0.489
Interval between completion of PCRT and surgical resection, days	52 (18–82)	54 (37–63)	0.633
mrTRG			0.222
1–2	79 (47.0)	13 (43.4)	
3–5	89 (53.0)	17 (56.6)	
Pathologic TRG of the primary tumor			0.001
Complete	55 (32.7)	1 (3.3)	
Near-complete	47 (28.0)	10 (33.4)	
Partial	34 (20.3)	16 (53.3)	
Poor or none	32 (19.0)	3 (10.0)	
Postoperative stage (Pathologic)			
ypT			0.008
ypT0	54 (32.1)	1 (3.3)	
ypT1	12 (7.1)	5 (16.7)	
ypT2	34 (20.3)	9 (30.0)	
ypT3+	68 (40.5)	15 (50.0)	
ypN			0.868
ypNx	10 (6.0)	2 (6.7)	
ypN(−)	120 (71.4)	20 (66.7)	
ypN(+)	38 (22.6)	8 (26.7)	

cRPV, combined response prediction value; AV, aval verge; PCRT, preoperative chemoradiotherapy; CEA, carcinoembryonic antigen; MRI, magnetic resonance imaging; TRG, tumor regression grade; mrTRG, magnetic resonance tumor regression grade.

## Data Availability

The data presented in this study are available on request from the corresponding author. The data are not publicly available due to the institutional policy.

## Supplementary Materials

The following are available online at https://www.mdpi.com/article/10.3390/cancers13143480/s1, Table S1: Clinicopathological characteristics of patients for whom the prediction using cRPV and mrTRG was incorrect, Table S2: Moderated regression analysis for cRPV’s prediction of pathologic response and the moderating effect of interval between completin of PCRt and follow-up MRI.
