# Supplementary material for: Improvement in the Assessment of Response to Preoperative Chemoradiotherapy for Rectal Cancer Using Magnetic Resonance Imaging and a Multigene Biomarker
Source: Cancers (Basel). 2021 Jul 12;13(14):3480. doi: 10.3390/cancers13143480 (PMC8305437; doi:10.3390/cancers13143480)
Supplement: Supplementary file 1 [file cancers-13-03480-s001.zip › cancers-1234301-supplementary.pdf]

cancers

MDPI

Supplementary Materials

Improvement in the Assessment of Response to Preoperative Chemoradiotherapy for Rectal Cancer Using Magnetic Resonance Imaging and a Multigene Biomarker

Eunhae Cho, Sung Woo Jung, In Ja Park, Jong Keon Jang, Seong Ho Park, Seung-Mo Hong, Jong Lyul Lee, Chan Wook Kim, Yong Sik Yoon, Seok-Byung Lim, Chang Sik Yu, and Jin Cheon Kim

Table S1. Clinicopathologic characteristics of patients incorrectly predicted by both cRPV and mrTRG.

Variables	No (%) or Median [Range]		p-Value
	Predicted by Either	Failed Prediction	
Age (years)	60 (32–89)	64 (39–86)	0.384
Sex			0.742
Female	64 (36.8)	8 (33.3)	
Male	110 (63.2)	16 (66.7)	
Tumor location from AV, cm	4.0 (0.3–10)	5.0 (1–10)	0.834
Pre-PCRT CEA, ng/mL	2.1 (0.3–115)	2.4 (0.60–11.3)	0.082
Pre-PCRT stage			
cT			0.644
cT3	154 (87.5)	22 (91.7)	
cT4	20 (11.5)	2 (8.3)	
cN			0.853
cN(−)	18 (10.3)	4 (13.7)	
cN(+)	156 (89.7)	20 (83.3)	
Chemotherapeutic regimen			0.231
Capecitabine	101 (58.0)	17 (70.8)	
5-fluorouracil/leucovorin	73 (42.0)	7 (29.2)	
Radiation dose, Gy	50.4 (43.2–50.4)	50.4 (45–50.4)	0.95
Post-PCRT CEA, ng/mL	1.6 (0.47–39)	1.5 (0.85–4)	0.447
Interval between completion of PCRT and follow-up MRI, days	41 (17–75)	43 (30–49)	0.109
Interval between completion of PCRT and surgical resection, days	52 (18–82)	54 (37–63)	0.196
Operative method			0.268
Local excision	11 (6.3)	3 (12.5)	
Radical resection	163 (93.7)	21 (87.5)	
mrTRG			0.263
1	33 (19.0)	9 (37.5)	
2	46 (26.4)	4 (16.7)	
3	56 (32.2)	8 (33.3)	
4	38 (21.8)	3 (12.5)	
5	1 (0.6)	0 (0)	
Pathologic TRG of primary tumor			0.015
Complete	55 (31.6)	1 (4.2)	
Near-complete	47 (27.0)	10 (41.7)	
Partial	40 (23.0)	10 (41.7)	
Poor or no	32 (18.4)	3 (12.5)	

Post-operation stage (pathologic)			
ypT			0.031
ypT0	54 (31.0)	1 (4.2)	
ypT1	13 (7.5)	4 (16.7)	
ypT2	35 (20.1)	8 (33.3)	
ypT3	65 (37.4)	11 (45.8)	
ypT4	7 (4.0)	0 (0)	
ypN			0.861
ypNx	10 (5.7)	2 (8.3)	
ypN(-)	123 (70.7)	17 (70.8)	
ypN(+)	41 (23.6)	5 (20.8)	

Abbreviations: cRPV=combined response prediction value; mrTRG = MRI tumor regression grade; AV = aval verge; PCRT = preoperative chemoradiotherapy; CEA = carcinoembryonic antigen; MRI = magnetic resonance imaging.

Table S2. Moderated regression analysis for cRPV's prediction of pathologic response and the moderating effect of interval between completin of PCRt and follow-up MRI.

Variables	Coef.	Standard Error	t
cRPV	0.893 ***	0.248	3.60
Interval between completion of PCRT and follow-up MRI (Interval), days	-0.001	0.004	-0.30
cRPV x Interval	-0.004	0.006	-0.74
Coefficient	0.184	0.178	1.03
R^2		0.502	
Adjusted R^2		0.495	
F (sig.)		63.950 ***	
N		194 †	

*** *p < 0.001.* cRPV = combined response prediction value; MRI = magnetic resonance imaging. † The number is 194 instead of 198 due to missing data on intervals between completion of PCRT and follow-up MRI.
